# T-Cell Engagers in Acute Myeloid Leukemia: Molecular Targets, Structure, and Therapeutic Challenges

**DOI:** 10.3390/cancers17193246

**Published:** 2025-10-07

**Authors:** Hunter Daws, Kate Gallinero, Amanda Singh, Sanela Bilic

**Affiliations:** 1 Vanadro Drug Development Consulting, West Des Moines, IA 50266, USA; 2 College of Pharmacy and Health Sciences, Drake University, Des Moines, IA 50311, USA

**Keywords:** acute myeloid leukemia, T-cell engagers, BiTE, DART, TandAb, DuoBody, DARPin, cytokine release syndrome, CD33, CD123

## Abstract

**Simple Summary:**

Acute myeloid leukemia remains difficult to treat due to its high relapse rates after standard therapies. T-cell engagers are a promising new approach that redirects the body’s own T cells to attack leukemia cells by binding both T cells and specific markers to AML cells. Targets such as CD33, CD123, and others have been studied. Several TCE formats—including BiTEs and DARTs—are being developed to improve effectiveness and reduce side effects. However, no TCEs in AML have moved beyond early-stage (Phase I/II) clinical trials, mainly due to the lack of an optimal target in AML and safety concerns with immune-related side effects like cytokine release syndrome. This review explores the current progress, key targets, and challenges in developing TCEs for AML treatment.

**Abstract:**

The treatment of acute myeloid leukemia (AML) remains challenging, largely due to high relapse rates following standard therapies. T-cell engagers (TCEs) offer a promising immunotherapeutic approach by redirecting T cells to recognize and kill AML cells. These therapeutic proteins bind CD3 to T cells and a tumor-associated antigen to AML cells, facilitating targeted immune activation. While CD33 and CD123 are the most commonly targeted AML antigens, others such as CD135, CD38, and CLEC12A/CLL-1 are being evaluated in preclinical and clinical studies. In parallel, various TCE formats—including BiTEs, DuoBodies, DARTs, and DARPin-based constructs—have been developed to optimize pharmacokinetics, stability, and immune engagement. Despite the growing number of TCEs entering clinical evaluation, none have advanced beyond early Phase (I/II) trials, primarily due to the lack of optimal target antigens and challenges in balancing antileukemic activity with the risks of immune-related toxicities such as cytokine release syndrome (CRS). This review aims to summarize the current landscape of TCE development in AML, highlighting key targets, formats, and challenges.

## 1. Introduction

Acute myeloid leukemia (AML) is a heterogeneous hematologic malignancy caused by the clonal expansion of myeloid blasts in the bone marrow and blood [1]. Typically, the incidence of AML is higher in adults aged 60 and older. AML therapies have improved in the last decade, but relapse is still common. First-line induction therapy generally leads to complete responses (CRs), though approximately 10–40% of younger patients and 40–60% of older patients are refractory to treatment. Hematopoietic stem cell transplantation (HSCT) is a second-line therapy for some patients, yet up to 40% of patients can still experience relapse [2]. Gemtuzumab ozogamicin (GO), a CD33-directed calicheamicin-linked antibody drug conjugate (ADC), is the only monoclonal antibody-based therapy approved for AML. Despite considerable efficacy in a subset of AML patients, concerns regarding the toxicity profile and benefit–risk ratio of GO point to an unmet need for additional immunotherapy options for the treatment of AML. Many new targets and novel approaches are being explored in this space, with the goal of taking advantage of disease-specific markers.

The development of novel immunotherapeutic approaches such as T-cell engagers (TCEs) for the treatment of AML has grown in recent decades and has rapidly expanded over the past several years.

TCEs are bispecific antibody-like structures that allow for simultaneous binding of an effector cell (i.e., T cell) and a tumor cell antigen, redirecting T cells against tumor cells by the formation of an immunologic synapse leading to T-cell-mediated cell killing. Despite the previous success of TCEs in the treatment of acute lymphoblastic leukemia (ALL) (i.e., blinatumomab), diffuse large B-cell lymphoma (DLBCL) (i.e., epcoritamab, glofitamab), and follicular lymphoma (FL) (i.e., mosunetuzumab), TCEs in AML have not been able to achieve similar clinical results. One reason for this is the lack of optimal target antigens in AML. For ALL and other B-cell malignancies, CD19, CD20, and CD22 provide ideal targets for immunotherapy, given that expression of these targets is restricted to the B-cell lineage. A similarly optimal target for AML has yet to be identified. The tumor cell antigens in AML that have been most frequently evaluated as TCE targets are the cell surface antigens CD33 and CD123. Other tumor cell antigens that are currently being evaluated in clinical studies include Fms-related tyrosine kinase (FLT3/CD135), CD38, and C-type lectin-like molecule-1 (CLL-1/CLEC12A).

The introduction of new and improved bispecific antibody (bsAb) formats has expanded the landscape of TCEs in AML beyond the traditional bispecific T-cell engager (BiTE) format, encompassing half-life extended (HLE) BiTEs, DuoBodies, dual affinity retargeting antibodies (DARTs), tandem diabodies (TandAbs), and designed ankyrin repeat proteins (DARPins). These next-generation TCEs have been designed with the goal of surmounting various challenges associated with TCE immunotherapy with increased stability, improved pharmacokinetic properties, and optimized affinities for target antigens and CD3. The affinities of the bispecific arms of a TCE to their respective target antigens are incredibly important in the context of safety, specifically the affinity for CD3. Some TCE development programs have opted to modulate the affinities of the bispecific arms to their respective targets, increasing affinity for the tumor-targeting arm and decreasing affinity of the CD3 targeting arm to further mitigate T-cell overactivation and associated immune-mediated toxicities.

A major challenge of TCE immunotherapy is the risk of immune-mediated toxicities, namely, cytokine release syndrome (CRS). CRS is a severe acute immune-mediated toxicity caused by the systemic release of cytokines. Clinically, CRS can present as fever, tachycardia, and hypotension and can escalate to multi-organ failure and death. TCEs are associated with an increased risk of CRS due to the secretion of pro-inflammatory cytokines such as IFNγ, TNFα, IL-2, and IL-6 by activated T cells as a part of the mechanism of action. Given that the appeal of TCEs is their potential for increased anti-tumor activity via immune system activation, identification of a safe and effective dose is critical for their development. In clinical trials evaluating TCEs, the mitigation of CRS has been achieved through the combination of various strategies. Premedication with corticosteroids and the use of anti-IL-6 mAb, tocilizumab, are two mitigation strategies that have been broadly utilized across clinical phase TCE development programs, as utilized with AMG330, JNJ-67571244, and many more. CRS risk is closely associated with the maximum concentration (C_max_) of a drug, particularly at the first dose. To reduce the risk of CRS, dose escalation strategies such as step-up dosing (also called priming dosing, or lead-in dosing) and/or different routes of administration (continuous IV infusion [cIV], subcutaneous [SC]) are often utilized for TCEs. Step-up dosing allows for the incremental increases of doses lower than the expected efficacious dose to prime the immune system, reducing the risk of cytokine storm. Selection of an optimal lead-in dosing schema is a large focus of early-phase clinical trials.

Although TCEs have proven to be clinically successful in other hematological malignancies, their place in AML is still being established. Previous and ongoing clinical trials have evaluated TCEs with a variety of different targets and structural characteristics. The aim of this review is to provide an overview of the current treatment landscape of TCEs in AML, as well as discuss the current challenges and future directions.

## 2. Overview of TCE Therapeutic Protein Formats

Following the example of FDA-approved blinatumomab, early TCEs in the AML space utilized a BiTE format. BiTE molecules are relatively small bsAbs (~55 kDa) that are formed by two single variable chain fragments (scFvs) covalently bonded to a single polypeptide linker. Notably, the classical BiTE format lacks a functional Fc domain and is thus unable to enact Fc-mediated effector functions, such as binding to the neonatal Fc receptor (FcRn) [3]. FcRn binding facilitates a saving mechanism via IgG recycling, preventing degradation and extending the half-life of the antibody [4]. Without the ability to bind to FcRn, traditional BiTE molecules have relatively short half-lives and may require administration via cIV to maintain adequate concentrations for sustained T-cell-mediated lysis of B cells [5]. In an effort to improve the short half-life of traditional BiTE molecules, HLE BiTEs were developed by fusing a traditional BiTE to an Fc domain, resulting in an IgG-like structure [6]. The addition of an Fc region to a BiTE construct has previously been demonstrated to result in a half-life of 210 h, as opposed to the half-life of a traditional BiTE molecule (~2–4 h) [6,7]. The Fc domain can also bind to the FcγR and enact immune-related Fc-mediated effector functions, such as ADCC, CDC, and ADCP, which can lead to immune-mediated cytotoxicity. Because these interactions are not necessary in the context of TCE therapy and can exacerbate CRS risk due to crosslinking of CD3 and FcγR, TCEs in recent development have mainly utilized a silenced Fc region that has been engineered via mutations such as L234A/L235A (LALA) to eliminate interactions with the FcγR [8,9]. An example of a TCE with this mutation is SAR440234.

DART molecules, another TCE format, are similar to BiTEs and utilize a diabody format composed of two scFv domains, one targeting CD3 and one targeting a tumor antigen, with the addition of a c-terminal disulfide bridge for increased stabilization. Like BiTEs, DARTs lack an Fc region [8]. MGD006 and MGD024 are two DART molecules detailed below in this review. TandAbs also utilize a diabody back bone similar to other fragment antibody formats such as BiTEs and DARTs, and comprise two scFvs for both the tumor target antigen and CD3 that are connected via a single polypeptide chain. They differ from previous TCE formats in that they are bivalent for each antigen. In TandAbs, the individual affinities of the tumor-targeting scFvs are relatively low compared to the other TCE formats; however, the two scFvs in tandem achieve stronger binding and increased avidity in areas where there is high expression of the tumor antigen. Additionally, TandAbs have the benefit of having a higher molecular weight that can exceed the renal clearance threshold [10]. AMV564, a TandAb, is detailed in this review.

The DuoBody format was developed in an effort to produce a bsAb format that retained the structure, function, and stability of an IgG1 molecule and allowed for the ability to utilize standard antibody discovery and production methods [11]. In the development of DuoBody TCEs, one Fab region from an IgG mAb targeting CD3 and one targeting the tumor antigen are exchanged in a process known as controlled Fab-arm exchange. This is possible via single match point mutations at the CH3 region of their respective Fc fragments that can promote heterodimerization [8]. JNJ-67571244 and JNJ-63709178 are two DuoBodies detailed in this review.

DARPins consist of tightly packed ankyrin repeats. Each repeat consists of thirty-three amino acids, with six forming a binding surface. DARPins are formed by two or three binding motifs contained between the N- and C-terminal motifs shielding hydrophobic regions. DARPins do not have an Fc region, are small (14–18 kDa), yet are highly stable [12]. DARPins are being exclusively developed by Molecular Partners, who are currently developing a CD3 x CD33 x CD123 x CD70 DARPin molecule, MP0533, in Phase 1. Other DARPin molecules are being studied in solid tumors and as a conditioning regimen for HSCT in AML.

Table 1 lists the formats, targets, and characteristics of TCEs evaluated in AML.

## 3. Challenges of TCEs in AML: Lack of Optimal Target Antigens

The potential success of TCEs in AML is limited by the lack of an optimal tumor-associated target antigen. Ideally, a target antigen in AML would be highly expressed in leukemic cells, with minimal expression in normal hematopoietic cells and other non-hematopoietic tissues. Such a target has yet to be identified due to the heterogeneity of AML and the shared expression of targets between AML blasts and normal hematopoietic cells [29]. The tumor antigens that have been the most extensively evaluated in the context of TCEs in AML are CD33 and CD123. Both CD33 and CD123 are heavily expressed in human AML cells, allowing for adequate targeting of leukemic cells. However, their expression in normal hematopoietic stem cells may lead to on-target off-tumor binding and increase the risk of hematologic toxicity when used as an immunotherapy target.

Both CD33 and CD123 are cell surface antigens confined to the myeloid lineage. Lineage-restricted antigens make up the majority of AML target antigens currently utilized in TCEs in development. Other lineage-restricted antigens that have been studied clinically in AML include CLEC12A/CLL-1, FLT3/CD135, and IL1RAP. Strong expression of a target antigen on leukemic stem cells (LSCs) is a desirable characteristic, given that LSCs have been hypothesized to be a reservoir for the recurrence of AML, due to their self-renewal capabilities and resistance to chemotherapy [30,31,32].

When considering immunotherapy targets, expression intensity, distribution, and localization are key characteristics to evaluate [33]. For therapeutic proteins such as TCEs, additional consideration should be given to the potential impact of broad expression on the pharmacokinetic profile. If a target is broadly expressed throughout the body, administration of a therapeutic protein at low doses may be inadequate to saturate the available target due to a phenomenon known as target-mediated drug disposition (TMDD). TMDD or “antigen sink” occurs when a large amount of the target is readily available to bind to the specific therapeutic protein format. When the therapeutic protein binds to the target, it is effectively cleared from the body. When there is a greater amount of target present in relation to the amount of therapeutic protein administered, the therapeutic protein will demonstrate non-linear pharmacokinetics characterized by rapid clearance as a result of binding and a significantly decreased half-life. This is why TMDD is most commonly observed at lower dose levels where saturation of the target has not been achieved. However, as dose levels increase, therapeutic protein concentrations are able to sufficiently saturate the available target, and a linear PK profile is observed. Interpatient variability in target expression and disease burden may influence the impact of TMDD on the PK profile and therefore influence the efficacy of a molecule or its associated toxicities [33].

### 3.1. CD33

CD33, also referred to as Siglec-3 (sialic acid binding Ig-like lectin 3), is a myeloid differentiation antigen that is widely expressed on human AML cells (>99%), though cell surface expression density is variable between patients. CD33 is expressed on AML blasts as well as LSCs and progenitor cells and has minimal expression in non-hematopoietic cells, making it an attractive target for AML immunotherapies [13]. In healthy individuals, CD33 is expressed in the early stages of myeloid cell development, with an estimated 30% of healthy bone marrow myeloid progenitor cells expressing CD33 [13,34,35]. Due to expression on normal hematopoietic cells, TCEs targeting CD33 bear a risk for on-target off-tumor binding and T-cell activation against healthy cells, which can lead to myelosuppression.

CD33 has been evaluated as a potential target for AML immunotherapies for several decades and is among the most extensively studied targets in this area. A large focus of CD33 as a target has been on ADC development. The approval of a CD33 targeted ADC, gemtuzumab ozogamicin (GO), validated CD33 as a target in AML; however, despite successful approval, several challenges have been recognized that limit its use in the clinic. These challenges include limited intracellular delivery of the cytotoxic payload in AML patients with relatively low expression of CD33 and the presence of drug transporter proteins that remove the cytotoxic payload from the cell prior to tumor cell killing. Notably, a Phase III clinical trial, SWOG S0106, found no overall improvement in addition to an increase in early deaths, and GO was temporarily withdrawn from market [35]. After further dose optimization, GO was re-approved in September 2017 at a fractionated dose of 3 mg/m^2^ days 1, 4, and 7, as opposed to the originally approved 9 mg/m^2^ on days 1 and 15. Due to the safety issues and the benefit to risk ratio of GO, alternative methods have been developed for targeting CD33, including immunotherapy options with improved efficacy in AML patients.

CD33 may be expressed as two different isoforms on the cell surface. The larger of the two isoforms possesses an intact V-set Ig-like domain, where the dominant epitope that is recognized by a majority of initial CD33 antibodies is located. Alteration of the CD33 structure to the shorter isoform that lacks the V-set Ig-like domain may impact the ability of CD33-targeting antibodies to recognize and bind CD33. Additionally, CD33 possesses endocytic properties when bound by bivalent antibodies, which can result in the internalization of antigen/antibody complexes, subsequently reducing cell surface expression. For ADCs such as GO, internalization is required for anti-tumor activity; however, internalization of CD33 may lead to loss of target and decreased binding when targeted with TCEs [36,37]. Despite the challenges observed with GO in the AML space, several TCEs targeting CD3xCD33 are currently being evaluated in ongoing clinical trials, as well as several that have been studied previously.

AMG330, developed by Amgen, is a CD33xCD3-targeted human BiTE antibody. AMG330 targets CD33 and the CD3ε chain on CD3, with its N-terminal and C-terminal scFvs, respectively. Preclinical in vitro studies evaluating AMG330 mediated lysis effectiveness at low effector–target (E:T) ratios, ranging from 1:3.4 to 1:79 [13]. During the development of AMG330, it was found that AMG330 did not modulate CD33 expression after continued exposure, unlike when CD33 is bound by a bivalent antibody such as GO [38]. Clinically, AMG330 was studied in a Phase 1 dose escalation study as a cIV infusion in 14- or 28-day cycles with a first-in-human (FIH) starting dose of 0.5 µg/day. As expected for a traditional BiTE molecule, AMG330 had a relatively short half-life of approximately 5 h. The most frequent treatment-related adverse events were CRS (78%), and this was mitigated with stepwise dosing of AMG330 and prophylactic dexamethasone and tocilizumab as needed. The maximum tolerated dose (MTD) was not reached, as the highest dose studied was 1600 µg/day. Complete response (CR), complete response with incomplete blood recovery (CRi), or morphologic leukemia free state (MLFS) were seen in 8/60 patients with a response assessment, and commonly between 7 of them had a target dose of 120–720 µg/day (NCT02520427) [39]. AMG330 has no active clinical trials.

AMG673 (Emerfetamab), developed by Amgen, is a half-life extended (HLE) BiTE targeted towards CD33 and CD3. While AMG673 is similar to AMG330, the key difference between the two molecules is the fusion of the N-terminus to a single-chain IgG Fc region on AMG673 [40]. Due to this, AMG673 had a longer half-life compared to AMG330 and did not require continuous IV administration. A Phase 1 dose escalation study evaluated AMG673 over a dose range of 0.05–110 µg IV, which was administered on Days 1 and 5 within a 14-day cycle. CRS was prevalent in 63% of the patients in the study. Dose-limiting toxicities (DLTs) occurred at the dose level of 110 µg, and the MTD was established as 72 µg. Although a reduction of blasts occurred in 42% of the patients in the study, only 1/33 patients achieved CRi (NCT03224819) [14]. AMG673 has no active clinical trials.

JNJ-67571244, developed by J&J, is a CD33xCD3 DuoBody with an Fc domain engineered with effector function-silencing mutations [15]. JNJ-67571244 binds to the C2 domain of CD33, which, unlike the V domain, is conserved in all CD33 isoforms as it is present closer to the cell surface and will not undergo single nucleotide polymorphism. Preclinically, JNJ-67571244 demonstrated potent cytotoxicity and T-cell recruitment in vitro at an E:T ratio of 1:5 [41]. JNJ-67571244 has been studied clinically in a Phase 1 dose escalation/expansion study, starting at a minimal anticipated biological effect level (MABEL) FIH dose of 0.2 µg/kg IV (twice weekly). Dose escalation reached a maximum treatment dose of 37.5 µg/kg via step up dosing. A once weekly SC administration arm was initiated at step-up doses of 0.63 and 2 µg/kg with a full treatment dose of 6.3 µg/kg on week three. The highest treatment dose level reached in the SC arm was 12.6 µg/kg. CRS of any grade was reported in 42.6% of the patients who received the study drug, which was mitigated with step-up dosing, premedication prophylaxis, and tocilizumab when indicated. Most patients in the study were unable to reach anticipated efficacious doses due to complexities required in the dosing schedule to combat CRS and other side effects, resulting in subtherapeutic concentrations. No patient in this study had an overall response better than stable disease, and the median overall survival was 4.1 months. In the IV administration groups, the half-life ranged from 63.7 to 100.2 h, whereas the SC half-life was not stated as it was abandoned after two dose-escalation cohorts (NCT03915379) [15]. JNJ-67571244 has no active clinical trials.

AMV564, developed by Amphivena Therapeutics, is a tandem diabody (TandAb) that targets CD3 and CD33. Preclinically, AMV564 successfully activated T cells at a concentration of 1 pM and E:T ratios as low as 1:10 [16]. AMV564 has been studied clinically in a Phase 1 dose escalation study, starting at an FIH of 0.5 µg/day via cIV. To mitigate CRS, prophylactic antiemetics, antipyretics, and antihistamines were administered, as well as a lead-in dosing escalation strategy. As a result, no patient had a Grade 3 or higher CRS event. Dose escalation reached 300 µg/day as the highest dose, with no DLTs. Three patients had either CR, CRi, or partial response (PR) (NCT03144245) [17]. AMV564 has a second Phase 1 study in monotherapy and in combination with pembrolizumab. AMV564 was administered via daily SC across the dose range of 5–75 µg/day. Preliminary results stated that clinically relevant exposures were achieved through SC administration, and clinical responses were seen in both monotherapy and combination arms (NCT04128423) [42]. Currently, no additional Phase 1 or Phase 2 clinical trials are active.

MP0533, developed by Molecular Partners, is a tetra-specific designed ankyrin repeat protein (DARPin) molecule, targeting CD3 on T cells, and CD33, CD123, and CD70 on AML cells. AML cells must express at least two of these antigens in order for MP0533 to bind, and healthy cells that express only one or none of these antigens will not be bound [43]. As of June 2025, MP0533 was evaluated in a Phase 1 dose escalation study, with the most frequent adverse event being CRS, occurring in 66% of patients, with three of those being grade 3 in severity. In total, 7 out of 41 patients achieved clinical response, 3 achieved MLFS, 2 achieved complete response with partial hematological recovery (CRh), and 1 achieved CR. Additionally, 13 out of 38 evaluable patients achieved a blast reduction of >50% in bone marrow. Notably, 3 subjects who achieved clinical response were in cohort 8 (n of 8), which utilizes an accelerated step-up dosing that adds an additional dose of MP0533 in cycle 1 (Day 12) (NCT05673057) [18]. No public information is available regarding the specific doses utilized in this study. This clinical trial is ongoing and is actively recruiting patients.

### 3.2. CD123

CD123, the low affinity binding alpha chain subunit of the IL-3 receptor, is a cell surface receptor expressed on cells of myeloid lineage, including myeloid progenitors, plasmacytoid dendritic cells, monocytes, and basophils [44]. Importantly, IL-3 has been identified as a cytokine that has been linked to AML cell proliferation [45]. CD123 is overexpressed in AML, though considerable variability and co-expression with mutations is seen across patients, with strong expression on both blast cells and LSCs in most AML patients, irrespective of a patient’s cytogenic or mutational profile [31]. CD123 expression at diagnosis in AML has been shown to be predictive of persistent measurable residual disease after induction therapy [46]. While the specific role of CD123 in leukemogenesis is currently unclear, overexpression of CD123 in AML is associated with a higher disease burden, increased blast proliferation, higher blast counts, and decreased overall survival [29,34]. Overall, CD123 overexpression on leukemic cells, high expression on LSCs, and its association with disease progression and resistance to chemotherapy provide ample reasoning to exploit CD123 as a target for AML immunotherapy. The N-extracellular domain of CD123 has been targeted with a number of molecules currently or recently in clinical stage studies, as discussed below.

MGD006 (Flotetuzumab), developed by MacroGenics, is a CD3xCD123 targeting dual-affinity retargeting antibody (DART). MGD006 was engineered to have a greater affinity for CD123 relative to CD3 to allow preferential binding and increased specificity for leukemic cells, thus decreasing the likelihood of off-target T-cell activation [19]. Preclinically, MGD006 was able to induce activation and expansion of T cells in vitro and T-cell-mediated killing of target cells in vitro and in vivo at low E:T ratios (<1:100) [47]. Given the potency of MGD006, cIV infusion was used as the route of administration in clinical studies to reduce the risk for CRS. In a FIH study, MGD006 was administered with an FIH starting dose of 3 ng/kg/day. Doses were escalated in single-patient dose escalation cohorts until MTD was reached at 700 ng/kg/day. In the Phase 1/2 study, the elimination half-life of MDG006 was determined to be 9.77 h at a dose of 500 ng/kg/day (NCT02152956) [48]. Currently, MGD006 has two active clinical trials, though neither is recruiting.

MGD024, developed by MacroGenics, is a second-generation CD3xCD123 DART molecule that has been designed to improve upon the characteristics of MGD006. MGD024 benefits from a prolonged half-life due to the incorporation of an engineered Fc domain that allows binding to FcRn but is mutated to silence binding to FcγR and complement (ala-ala-mutated human IgG1 Fc). MGD024 also benefits from a mutation on the CD3 targeting arm that engineers the molecule to have further decreased affinity for CD3, reducing the risk of cytokine release compared to the previous MGD006 molecule. The improvements made for MGD024 allow for intermittent (weekly or longer) administration, as opposed to administration via cIV infusion that was previously used for MGD006. In preclinical studies, MGD024 showed reduced potency and reduced cytokine release compared to MGD006 in in vitro and in vivo models; however, higher doses of MGD024 were able to achieve magnitude of tumor growth reduction as observed with MGD006 [20]. MGD024 is currently being studied in a Phase 1 FIH dose escalation study in patients with relapsed/refractory (R/R) CD123+ hematologic malignancies including AML (NCT05362773) [49].

XmAb14045 (Vibecotamab), developed by Xencor, is a CD3xCD123 targeting molecule created with the XmAb bispecific platform. The XmAb platform allows for efficient development of bsAbs and Fc fusions, producing antibodies with a heterodimeric Fc that can support FcRn binding for half-life, effector functions (if warranted), and mixed valency of the TAA and CD3 arms. The Fc domain of XmAb14045 has been engineered to silence interactions with FcγR, allowing for the advantage of maintaining a long serum half-life through preserving binding capabilities to FcRn while reducing the potential for nonselective T cell activation [21]. Preclinically, XmAb14045 induced CD123+ AML cell death at approximately 1 ng/mL. In cynomolgus monkeys, it was demonstrated that XmAb14045 could achieve CD123+ depletion not only in the circulation but also in bone marrow [50]. A clinical Phase 1 dose escalation study was conducted and completed, examining IV dosing across the dose range of 0.003 to 12 µg/kg. Several step-up dose strategies were utilized, ranging from once weekly, two times per week, three times per week, and four times per week, dosing during the first week of treatment. Premedication, in addition to the step-up dosing, was used to mitigate CRS, though 102/120 patients experienced CRS, with 32 of those being grade 3 or higher. Regarding clinical response, 10 patients achieved either a CR (3), CRi (3), MLFS (3), or PR (1). It was noted that patients who had clinical responses had a statistically significant lower absolute blast count (<25%) at baseline and received target doses of >0.75 µg/kg. The half-life was shorter than anticipated and ranged from 10 to 25 h. A recommended Phase 2 dose (RP2D) schedule was achieved (3 times per week step-up dosing in the first week: 0.43, 0.75, 1.1 µg/kg, followed by weekly 1.7 µg/kg) (NCT02730312) [51]. XmAb14045 is currently in a Phase 2 study examining utility in minimal residual disease at the M.D. Anderson Cancer Center (NCT05285813).

JNJ-63709178, developed by J&J, is a CD3xCD123 bispecific humanized IgG4 antibody generated by a Fab-arm exchange, exclusively by Genmab DuoBody technology. Preclinically, JNJ-63709178’s analog, CNTO9958, was able to induce depletion of leukemic blasts at E:T ratios as low as 1:54.3 [22]. In the Phase 1 dose escalation study, both IV and SC routes of administration were examined. In the IV cohorts, the dose range examined was 0.15–6 µg/kg, with frequences of every two weeks (Q2W) and twice weekly. In the SC cohorts, the dose range examined was 0.3–4.8 µg/kg twice weekly. Varied step-up dosing was utilized throughout the study to mitigate CRS, and overall, CRS totaled 43.5% of patients across all cohorts. The mean half-life in the 4.8 µg/kg IV cohort was 39.6 h. Ultimately, unfavorable safety in both IV and SC cohorts prevented the higher dosing needed to achieve exposures within a therapeutic window. T-cell engagement was present, although no relevant clinical efficacy was achieved, as only one patient achieved stable disease during the study. Dose-escalation did not find RP2D (NCT02715011) [52]. JNJ-63709178 has no active clinical studies.

APVO436, developed by Aptevo Research and Development LLC, is a CD3xCD123 targeting ADAPTIR molecule. The structure of ADAPTIR molecules consists of two sets of binding domains that are linked via a modified human IgG Fc domain engineered to minimize binding to the FcγR. In vitro, APVO436 was able to demonstrate the ability to induce cytotoxicity at low E:T ratios and induce lower levels of cytokines associated with CRS compared to MGD006 [23]. In a Phase 1/1b FIH study, APVO436 was tested in R/R AML patients starting across the dose range of 0.3 to 60 µg via weekly IV infusions. RP2D was found, with a cumulative cycle 1 dose of 54 µg: first dose 6 µg, second dose 12 µg, third and fourth dose 18 µg, followed by 18 µg weekly dosing. CRS was the most common AE, occurring in 21.7% of patients. At the RP2D level, two patients had CR, plus one patient with myelodysplastic syndrome (MDS) achieved marrow CR, while two patients had progressive disease (PD) and the rest achieved stable disease (SD) (NCT03647800) [53]. A new Phase 1b/2 study in patients with newly diagnosed AML and in combination with venetoclax and azacitidine is currently ongoing (NCT06634394).

SAR440234, developed by Sanofi, is a CD123xCD3-targeted TCE that uses a bispecific cross-over dual-variable domain (CODV) format [24]. The engineering of SAR440234 included the fusion of a bispecific CODV-Fab module to an IgG1-Fc region that contains a LALA double mutation to reduce Fc effector function. Preclinically, T-cell activation and cytotoxicity were examined under an E:T ratio of 10:1 and exhibited picomolar EC50 in CD123-positive AML cells. Interpatient variability in E:T ratios was observed in AML patient samples, ranging from 1:1 to 1:100, though there was no impact on the activity of SAR440234 [54]. A Phase 1 dose escalation study to evaluate SAR440234 administered via IV infusion in patients with AML, ALL, and myelodysplastic syndrome (MDS) was being conducted; however, the study was terminated with no publicly available information on the study results (NCT03594955).

### 3.3. FLT3

Fms-related tyrosine kinase (FLT3), also referred to as CD135, is a type 1 transmembrane protein and lineage-associated growth factor that plays an essential role in hematopoiesis. In normal hematopoietic cells, expression of FLT3 is restricted to CD34+ stem and progenitor cells, including myeloid, lymphoid, and dendritic cell progenitors. In AML, FLT3 is expressed in both blast cells and LSCs and is overexpressed in 89% of patient AML bone marrow samples compared to bone marrow controls [55,56,57]. FLT3 mutations occur in approximately 30% of AML patients and include internal tandem duplications and point mutations in the tyrosine kinase domain. These mutations are associated with poorer prognosis, higher relapse rates, and reduced survival rates. Tyrosine kinase inhibitors (TKIs) targeting mutant FLT3 have been evaluated clinically in AML patients, with some success, including FDA approval of Midostaurin for newly diagnosed FLT3-mutated AML in combination with chemotherapy. However, the activity of FLT3 TKIs is dependent on the presence of mutant FLT3, whereas targeting FLT3 with a TCE would allow for targeting regardless of mutational status [57].

AMG427, developed by Amgen, is a half-life extended BiTE molecule that targets FLT3 and CD3, comprising binding domains for FLT3 and CD3 fused to the N-terminus of a Fc region. AMG427 bound to human FLT3 and CD3, with affinities of 0.5 nM and 8.2 nM, respectively. Preclinically, AMG427 demonstrated picomolar potency against human FLT3-positive cell lines inducing cytotoxicity, with mean EC50 values ranging from 1.1–3.0 pM. Of note, it was concluded that in vitro efficacy was improved in samples with higher FLT3 expression and an E:T ratio > 1:38 [26]. Clinically, a Phase 1 study evaluating the safety and efficacy of AMG427 was being conducted; however, the study and program were terminated prematurely with no results available publicly (NCT0354136).

CLN-049, developed by Cullinan Therapeutics, is an anti-FLT3xCD3 bsAb constructed as an IgG heavy chain/scFv fusion. The Fc region on CLN-049 was silenced, ensuring no interaction with FcγR. In preclinical in vitro assays, CLN-049 was studied for its ability to induce lysis of AML blasts at physiological E:T ratios from AML patient samples. It was found that CLN-049 sufficiently induced lysis across the E:T ratios 1.6:1, 0.42:1, and 0.3:1 [27]. Clinically, CLN-049 is being evaluated in an ongoing Phase 1 dose escalation study (NCT05143996).

### 3.4. CLL-1/CLEC12A

C-type lectin-like molecule-1 (CLL-1), also known as c-type lectin domain family 12 member A (CLEC12A), is a type II transmembrane glycoprotein and a myeloid differentiation antigen restricted to myeloid lineage cells [58]. CLEC12A is expressed in 90–95% of AML patients, with broad expression in LSCs and AML blasts [25,59]. Importantly, CLEC12A demonstrates selective expression on LSCs, with no expression on normal hematopoietic stem and progenitor cells, reducing the risk for myelosuppression for CLEC12A targeting immunotherapies compared to immunotherapies targeting CD33 and CD123 [25,58]. The favorability of this lack of expression within the normal stem/progenitor cell compartment contributes to the interest in CLEC12A as a target in AML. However, CLEC12A is expressed in normal immature and mature myeloid cells, including common myeloid progenitors (CMPs), granulocytes, macrophages, and monocytes. Thus, TCEs targeting CLEC12A may induce T-cell killing against these cell populations, increasing the risk of infection [58]. CLEC12A has previously been evaluated as an immunotherapeutic target with ADCs [58,60,61]. The development of the first CLECL12A targeting TCE is described below.

MCLA-117, developed by Merus N.V., is a human full-length CLEC12AxCD3 targeting IgG1 bispecific antibody. MCLA-117 was engineered to have increased affinity for CLEC12A relative to CD3 (3 nM vs. 117 nM, respectively) [62]. The presence of an intact Fc region on MCLA-117 provides the benefit of an extended half-life via FcRn-mediated recycling. Additionally, incorporation of amino acid substitutions on the CH2 region allows for silencing of Fc interactions with FcγR, without impacting the ability to interact with FcRn [62,63]. In preclinical in vitro studies, MCLA-117 was observed to induce T-cell-mediated lysis of AML blasts in primary AML bone marrow samples and was evaluated across E:T cell ratios ranging from 1:7 to 1:80. MCLA-117 did not bind to normal hematopoietic stem cells (HSCs) or common myeloid progenitor cells, as expected, given the expression profile of CLECL12A [25]. A Phase 1 study evaluated the dose escalation and expansion of MCLA-117 in adult patients with AML. Preliminary results found that 36.2% of patients experienced CRS, which was mitigated by administration of prophylactic antihistamines and tocilizumab when indicated. Dose escalation utilized a ramp-up scheme, where increasing flat IV doses were administered, followed by a weekly infusion at the target dose level (range 0.675–240 mg). The first two cohorts were given priming doses on Days 1, 3, 5, 8, 11, 15, and 22. At the highest target dose level tested (240 mg), priming doses of 5, 15, and 25 mg were administered on days 1, 4, and 8. Clinical activity was observed via blast reduction of >50%; however, only 1 patient achieved MLFS. The development of MCLA-117 did not proceed into the dose expansion cohorts of Phase 1 due to the lack of observed clinical activity (NCT03038230) [63].

### 3.5. CD38

CD38 is a class II transmembrane glycoprotein on the cell surface previously established as a target in multiple myeloma (MM) and T-cell acute lymphoblastic leukemia [64]. Antibody-based therapies targeting CD38 have been previously successful in MM, as evidenced by the clinical efficacy of daratumumab, isatuximab, and TAK079. CD38 is predominantly expressed in lymphocytes and myeloid cells, with a high expression in early and activated cells and a low expression in mature cells. In AML, there is significant variation in the cell surface expression of CD38 (58.2% to 91.7%) [65,66]. CD38 has been viewed as a suboptimal target in AML, as a majority of LSCs are CD38 negative. However, it has been shown in vitro that in the presence of IFN-γ, CD38 expression is induced via the conversion of CD34+, CD38− LSCs into CD34+ CD38+ blasts, providing targetable CD38 expression on the previously CD38- LSCs [67]. Thus, the use of CD38-directed immunotherapy has potential efficacy. The development of a CD38-targeting TCE is discussed below.

XmAb18968, developed by Xencor, is a CD3xCD38 targeting molecule that utilizes an XmAb format. Similar to the previous XmAb molecule discussed previously (see XmAb14045), XmAb18968 has an Fc domain that is engineered to minimize interactions with FcγR, thereby reducing non-selective T-cell activation and cytokine release. In a Phase 1 dose escalation study, XmAb18968 was studied at dose levels of 0.8, 1, 1.3, and 1.5 mg administered via IV infusion on days 1, 8, 15, and 22 on a 28-day cycle. CRS was observed in 62% of patients, with a mitigation plan for step-up dosing on days 1 and 2. No DLTs were noted during the study, and two patients out of 13 achieved minimum residual disease (MRD) negative CR. The two patients who achieved CR then proceeded to allogeneic HCT (NCT05038644) [28]. The Phase 1 study has since been terminated, and there are currently no active clinical trials evaluating XmAb18968 being conducted.

Table 2 presents the available clinical data of TCEs in AML.

## 4. Future Directions

Continued search towards new targets specific to AML that minimize normal tissue and cell involvement should be the mainstay of future TCE development in AML. Furthermore, drug developers are investing in the development of molecules that are able to bind to intracellular targets. Thus far, most TCE development programs in the AML space have targeted cell surface proteins [33]. However, in the past few years, TCE molecules targeting intracellular targets have emerged, opening up new possibilities for identifying an optimal target antigen in AML. TCR-mimetic (TCRm) TCEs utilize the highly specific T-cell receptor (TCR) to bind to intracellular antigens that are presented on the cell surface by major histocompatibility complex (MHC) molecules with high affinity. TCR-based therapies have had previous success, as evidenced by the FDA approval of tebentafusp, a TCR-CD3 bispecific molecule with a TCR specific for gp100 [68]. In addition, optimization of the respective binding affinities for each arm of the TCE has been explored in previous TCE development programs in an effort to balance the potency and safety of the molecule.

As discussed previously, the lack of optimal target antigens is a significant barrier to the success of TCEs in AML. An additional target overexpressed in AML blasts and LSCs, IL1RAP (interleukin-1 receptor accessory protein), has emerged with promising preclinical data [69,70]. IL1RAP is expressed in normal hematopoietic stem and progenitor cells, but previous studies have established that targeting IL1RAP does not influence normal hematopoietic cells [71]. Additionally, IL1RAP demonstrates lower expression on HSCs compared to other AML cell surface antigens previously targeted in the clinic (i.e., CD33, CD123, and CLEC12A) [69]. BiF-002, an IgG-like TCE specific for CD3xIL1RAP, is reported to be the first IL1RAP targeting TCE, though IL1RAP has been studied previously as an immunotherapeutic target in the context of solid tumor, chronic myeloid leukemia (CML), and AML therapies. BiF002 was developed using Fab arm exchange and exhibits a strong affinity to IL1RAP with a relatively weak affinity to CD3. BiF002 has a mutated human IgG1 Fc domain that silences FcγR binding. As expected, due to the low expression of IL1RAP in healthy hematopoietic cells, BiF002 was not observed to impact normal hematopoiesis in preclinical in vitro studies, thus providing encouraging evidence of IL1RAP as a potential optimal target antigen for TCEs in AML [72]. BiF-002 has yet to be evaluated in clinical trials.

ABBV-184, developed by Abbvie, is a survivin (baculoviral inhibitor of apoptosis repeat-containing 5; BIRC5) peptide-specific TCRxCD3 TCRm TCE. ABBV-184 binds a peptide derived from survivin that is bound to the class I MHC (HLA)-A*02:01 expressed on the surface of tumor cells [73]. Survivin is highly expressed in solid tumors and hematologic malignancies, including AML, with significantly higher on CD34 + CD38− LSCs compared to bulk AML blasts (*p* < 0.05) [74]. ABBV-184 was previously evaluated in a FIH Phase 1 dose escalation study in patients with AML and non-small cell lung cancer (NSCLC) with an HLA-A2:01 restricted genotype (NCT04272203). In this study, 15 patients (8 AML and 7 NSCLC) were enrolled, and dose escalation ranged from 0.07 to 0.7 µg/kg. Transient elevations in cytokines were observed at all dose levels, and Grade 1–2 IRR and CRS TEAEs were reported. The reported ABBV-184 half-life was approximately 13–29 h [75]. The study has since been terminated.

CBX-250, developed by Crossbow Therapeutics, is a TCRm TCE that targets cathepsin G peptide (CG1)/HLA-A2*02:01xCD3 [76]. CG1 is an intracellular serine protease that is endogenously expressed in cells of myeloid lineages, with particularly high expression in AML blasts and LSCs compared to normal HPCs [77]. CBX-250 binds to CG1/HLA-A2*02:01 pMHC complex with high affinity [76]. CBX-250 is currently being evaluated in an ongoing Phase 1 dose escalation trial (NCT06994676) in patients with AML, High-Risk (MDS), or Chronic Myelomonocytic Leukemia (CMML). CBX-250 is to be administered via SC administration in 28-day cycles. In Cycle 1, a priming phase will occur over 7 days, with a target phase over 28 days (ClinicalTrials.gov). Currently, no data has been published on this study. Despite the termination of the ABBV-184 Phase 1 trial, the development of CBX-250 and ABBV-184 into the clinical space represents a new avenue for identification of an optimal target antigen in AML.

An example of a TCE in lymphoma is Glofitamab, a bivalent TCE approved for R/R DLBCL that utilizes a 2+1 format, where one arm has two binding sites for CD20 and one arm specific for CD3 [78]. The presence of two tumor-binding domains allows for highly specific tumor binding, avidity, and improved on-target T-cell activation at the site of action. A TCE utilizing this format has yet to be evaluated clinically in AML; however, the success of Glofitamab in DLBCL points to a potential benefit in the application of this TCE format in other hematologic malignancies.

## 5. Conclusions

The development of TCEs remains an area of focus within the AML space. Although many TCEs have made it to clinical trials in AML, challenges with balancing safety and efficacy have arisen, limiting potential clinical success. These challenges include the lack of a suitable target antigen, a short half-life, and mitigation of CRS and other toxicities. A variety of tumor antigens have been targeted in AML, expanding from CD33 and CD123 to include CLEC12A, FLT3, and CD38. Currently, no single-cell surface antigen has separated itself as an optimal target in the AML space based on clinical efficacy. However, there is encouraging preclinical data with a TCE targeting IL1RAP, as well as the emergence of TCRm TCEs specific for intracellular targets.

There have been continuous improvements in the development of TCEs in AML, motivated by the desire to improve the risk–benefit profile associated with these molecules. Strategies to optimize the pharmacological characteristics of TCEs have been explored via the development of improved therapeutic protein formats, Fc engineering, and affinity optimization. These novel improvements in TCE drug development show encouraging preliminary data on safety and efficacy to be able to strive towards the goal of bringing additional clinically successful molecules to patients.

## Figures and Tables

**Table 1:** Formats, targets, and characteristics of T-cell engagers evaluated in acute myeloid leukemia.

Target	Molecule	Format	Intact Fc Region	Fc Mutations	References
CD33	AMG330	BiTE	No	N/A	[13]
	AMG673	HLE BiTE	Yes	Not disclosed	[14,15]
	JNJ-67571244	DuoBody	Yes	FcγR silenced	[16]
	AMV564	TandAb	No	N/A	[17]
	MP0533	DARPin	No	N/A	[18]
CD123	MGD006	DART	No	N/A	[19]
	MGD024	Second generation DART	Yes	FcγR silenced	[20]
	XmAb14045	XmAb	Yes	FcγR silenced	[21]
	JNJ-63709178	DuoBody	Yes	FcγR silenced	[22]
	APVO436	ADAPTIR	Yes	FcγR silenced	[23]
	SAR440234	CODV	Yes	FcγR silenced	[24]
CLEC12A	MCLA-117	Full length IgG1	Yes	FcγR silenced	[25]
FLT3	AMG427	HLE BiTE	Yes	FcγR silenced	[26]
	CLN-049	IgG/scFv fusion	Yes	FcγR silenced	[27]
CD38	XmAb18968	XmAb	Yes	FcγR silenced	[28]

**Table 2:** Available clinical data.

Molecule (Target)	Development Phase	Dose Escalation and Route	CRS	Efficacy	Reference
AMG330 (CD33)	Phase I—Terminated (NCT02520427)	cIV; Dose range of 0.5–1600 µg/day	78% of patients had any grade CRS	8/60 patients had CR, CRi, or MLFS	[40]
AMG673 (CD33)	Phase I—Terminated (NCT03224819)	IV; Dose range of 0.05–110 µg	63% of patients had any grade CRS	1 patient achieved CRi	[14,15]
JNJ-67571244 (CD33)	Phase I—Completed (NCT03915379)	IV: Dose range of 0.2–37.5 µg/kg SC: Dose range of 0.63–6.3 µg/kg	42.6% of patients had any grade CRS	No patient had a response better than stable disease	[41]
AMV564 (CD33)	Phase I—Completed (NCT04128423)	cIV; Dose range of 0.5–300 µg/day	No incidence of grade 3 or higher CRS	3/36 patients had either a CR, CRi, or PR	[43]
MP0533 (CD33)	Phase 1—Ongoing (NCT05673057)	N/A	66% of patients had any grade CRS; 3 cases of grade 3	7/41 achieved clinical response: 3 MLFS, 2 CRh, 1 CR 13/38 patients achieved a blast reduction of ≥50% in bone marrow	[44]
MGD006 (CD123)	Phase I—Terminated (NCT02152956)	cIV: Dose range of 3–700 ng/kg/day	N/A	N/A	[48]
MGD024 (CD123)	Phase I—Ongoing (NCT05362773)	N/A	N/A	N/A	
XmAb14045 (CD123)	Phase 1—Complete (NCT02730312) Phase 2—Active, not recruiting (NCT05285813)	IV: Dose range of 0.003 to 12 µg/kg	102/120 patients had any grade CRS; 32 being grade 3 or higher	10/120 clinical responses: 3 CR, 3 CRi, 3 MLFS, 1 PR	[51]
JNJ-63709178 (CD123)	Phase 1—Complete, no active trials (NCT02715011)	IV: Dose range of 0.15–6 µg/kg SC: Dose range of 0.3–4.8 µg/kg	43.5% had any grade CRS	One patient achieved SD	[52]
APVO436 (CD123)	Phase 1b/2—Active (NCT06634394)	IV: Dose range of 0.3–60 µg	21.7% had any grade CRS	At RP2D, two patients had CR, two had PD, and the rest had SD	[53]
SAR440234 (CD123)	Phase 1—Terminated (NCT03594955)	N/A	N/A	N/A	
MCLA-117 (CLEC12A/CLL-1)	Phase 1—Terminated (NCT03038230)	IV: Dose range of 0.025–240 mg	36.2% had any grade CRS	1 patient achieved MLFS	[63]
AMG427 (FLT3)	Phase 1—Terminated (NCT0354136)	N/A	N/A	N/A	
CLN-049	Phase 1—Ongoing (NCT05143996)	N/A	N/A	N/A	
XmAb18968 (CD38)	Phase 1—Terminated (NCT05038644)	IV: Dose range of 0.8–1.5 mg	62% had any grade CRS	2/13 achieved MRD negative CR and proceeded to allogeneic HCT	[28]

## Data Availability

No new data were created or analyzed in this study. Data sharing is not applicable to this article.
